# Ultrawideband, Wide Scanning Stripline-Fed Tightly Coupled Array Antenna Based on Parallel-Dipole Elements

**DOI:** 10.3390/s20185065

**Published:** 2020-09-06

**Authors:** Xiuye Liang, Weishuang Yin, Ang Chen, Zhe Zhang, Jianping Zeng, Lei Shi, Fang Guan, Xiaohan Liu, Jian Zi

**Affiliations:** 1 Department of Physics, Fudan University, Shanghai 200438, China; xyliang16@fudan.edu.cn (X.L.); wsyin14@fudan.edu.cn (W.Y.); achen12@fudan.edu.cn (A.C.); zzhang17@fudan.edu.cn (Z.Z.); jpzeng18@fudan.edu.cn (J.Z.); lshi@fudan.edu.cn (L.S.); liuxh@fudan.edu.cn (X.L.); jzi@fudan.edu.cn (J.Z.); 2 Institute for Nanoelectronic Devices and Quantum Computing, Fudan University, Shanghai 200438, China

**Keywords:** ultrawideband (UWB), phased array antenna, tightly coupled dipole array (TCDA), cross-polarization, wireless sensing

## Abstract

A stripline-fed tightly coupled array antenna with compact size, large scan volume and low cross-polarization characteristics is proposed for ultrawideband (UWB) applications. Simple impedance-matching process is realized by using parallel dual dipoles. Meanwhile, the parallel symmetrical radiating structures minimize the cross-polarization field components dramatically. The mitigation of various undesired resonances is studied in detail. An infinite array is designed to achieve 3:1 bandwidth (6−18 GHz) when scanning ±60${}^{\circ }$ in the E-/D-planes (VSWR < 2.5) and H-plane (VSWR < 3.5). The cross-polarization levels remain below −29 dB at broadside. A 16 × 16 prototype is fabricated to demonstrate the design. The measured results are consistent well with the simulated ones. The overall size of the prototype at the lowest operating frequency is $3\;\times 3\;\times 0.4\;\lambda_{0}^{3}$ ($15\;\times 15\;\times 2\;{\mathrm{cm}}^{3}$). Due to its wide bandwidth, good electronic scan performance and compact size, the proposed antenna array is a good candidate for modern wireless platforms.

## 1. Introduction

Modern wireless electronic platforms are expected to have advanced sensors to cover wide frequency band and/or large scanning range. The ultrawideband array antennas with large scan volume and compact size are essential components of these systems, such as 5G multiple-input–multiple-output (MIMO) communication systems [1,2,3], radio telescopes [4,5] and high resolution radars and imagers [6,7,8]. These UWB arrays can realize multiple functions within one single radiating aperture achieving a significant reduction of the size, weight, cost and power consumption.

The Vivaldi (tapered slot or flared notch) radiator is one of the most popular elements in UWB phased arrays [9,10,11]. This type of array can typically achieve 10:1 impedance bandwidth with ±60${}^{\circ }$ scan angle. However, they can be several wavelengths thick and suffer from high cross-polarization while scanning [12]. In recent years, a novel class of arrays referred to as connected dipole arrays (CDAs) [13,14,15,16] or tightly coupled dipole arrays (TCDAs) [17,18,19] was proposed. They have emerged as attractive options in UWB systems, due to their wide bandwidth, wide-angle scanning, low profile, and low cross-polarization characteristics. The operating principle of this kind of array is fundamentally different from that of the traditional antenna arrays. Usually, isolated elements are employed in an antenna array and the mutual coupling effect between array elements is undesirable. While in the CDAs or TCDAs, dipole elements are spaced very close to each other and strong mutual coupling is artificially introduced, thus realizing the continuous sheet current from Wheeler [20]. There is a slight difference between the CDA and the TCDA. In TCDA, the inter-element capacitive coupling is introduced to counteract the ground plane inductance, thereby achieving larger antenna bandwidth [21].

To practically implement a TCDA, the array feeding network and impedance matching are critical. Since the high impedance of the TCDA aperture ($Z_{in}\approx$ 377 Ω), it is hard to match with the standard 50 Ω coaxial interfaces directly. Researchers have proposed many practical feeding architectures, such as feed organizers [22], coaxial cable feeds [23], TCDA with integrated baluns (TCDA-IB) [24,25,26,27,28], Planar Ultrawideband Modular Array (PUMA) feeds [29,30,31]. Generally, aiming at the impedance matching, a dielectric superstrate is usually placed over the dipole elements to drop the aperture impedance from 377 Ω down to 200 Ω. The use of the bulky dielectric layer will surely increase the weight, cost and fabrication complexity for the total array, and may incur detrimental surface waves. In [32,33,34,35,36], a lightweight frequency selective surface (FSS) or a metasurface instead of the dielectric superstrate was used to improve wide-angle scanning. However, they still suffer from long multi-section feeding lines. The stepped feedlines used as the impedance transformers are usually obtained from brute-force optimization, and may be not easily scaled to very high frequencies due to the machining tolerances.

In this paper, a novel stripline-fed tightly coupled array based on parallel-dipole elements is proposed. The parallel combination of the dual dipoles reduces the aperture impedance by a factor of almost four, thus resulting in simple impedance matching with a typical 50 Ω coaxial line. This configuration eliminates the need for the bulky dielectric superstrate or the multi-section stepped transformer, providing a simple, low-cost and lightweight array structure. Good cross-polarization performance is realized by using the double-sided symmetrical dipoles. The cross-polarization levels remain below −33 dB and −8.5 dB for scanning to broadside and 60${}^{\circ }$ in D-plane, respectively. Instead of the microstrip, the stripline feeding network combining with the plated-through vias is used to avoid the energy leakage and mitigate the undesired resonances. Moreover, a printed FSS is employed to improve matching and scanning. A single-polarized array operating over 6−18 GHz (3:1) is designed to achieve VSWR < 2.5 when scanning to 60${}^{\circ }$ in the E- and D-planes, and VSWR < 3.5 when scanning to 60${}^{\circ }$ in the H-plane. Good agreement is obtained between measured and simulated results. As a phased array, the antenna demonstrates compact size, low profile and good scanning performance, making it a superior candidate for UWB multi-function radio frequency (RF) systems.

## 2. Array Design and Simulation

**Figure 1:**
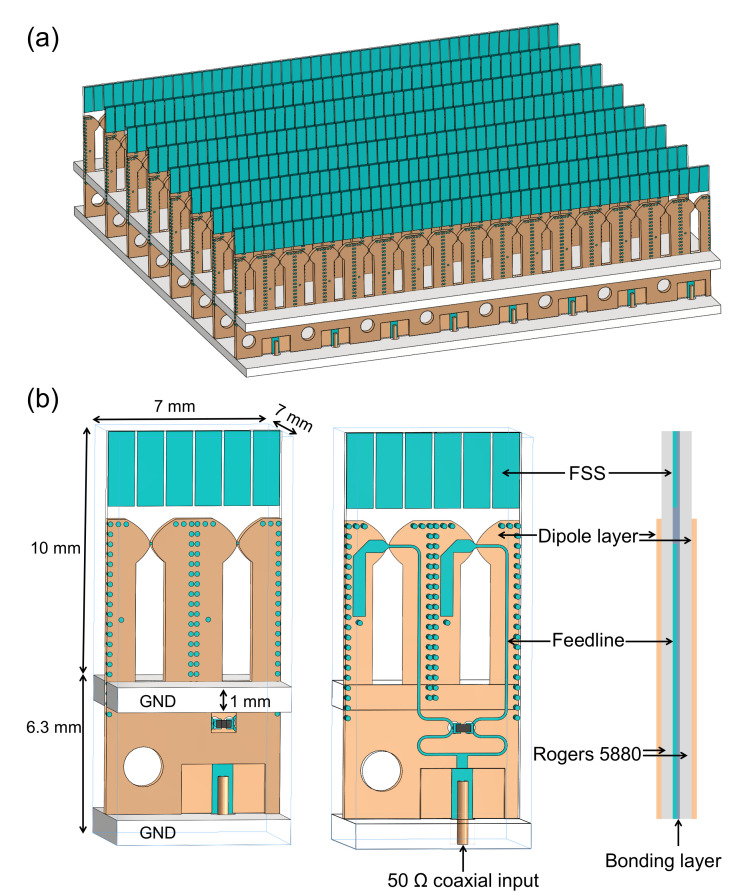
Topology of the proposed TCDA. (**a**) 8 × 8 array; (**b**) Unit cell: exterior view, interior view (with the front dipole layer removed), and side view.

The proposed TCDA with feeding networks and FSS superstrate is shown in Figure 1a. The element configuration is presented in Figure 1b, which is implemented using double-layer PCB laminating technology. Three copper layers are hosted by two Rogers 5880 (ϵ=2.2) substrates of 10 mil thickness each. These layers are laminated together using a 4-mil-thick polyflon material. Two dipole layers are printed on the opposite sides of the substrate, and the FSS together with the feedline is placed in the middle layer. Such sandwich structure constitutes a stripline feeding network. The array consists of two radiators per unit cell, each of which contains a pair of parallel dipoles. As emphasized later, it is critical for the impedance transformation of the array. Two ground planes are employed, the top one serves as a reflector for the dipoles, and the bottom one is used to hold the coaxial connectors. The distance between them should be small enough to produce a low profile.

**Figure 2:**
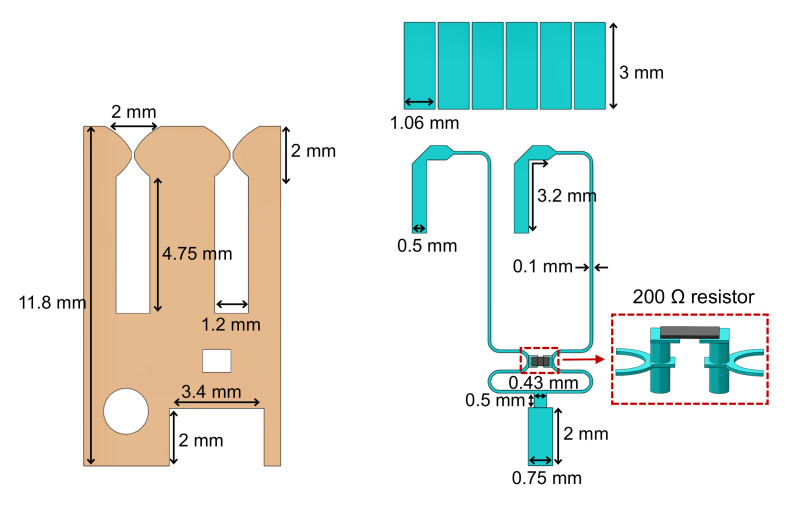
Detailed geometry of the proposed TCDA periodic unit cell (including the front dipole layer, FSS, Marchand balun and Wilkinson divider).

Figure 2 illustrates key details of the front dipole layer and the feeding network, including basic dimensions. An exponent-shaped dipole is chosen as the element itself has good radiation characteristics. The feeding structure is composed of a Marchand balun and a Wilkinson divider. To facilitate the installation of the isolation resistor, a slot is cut in the front metal layer. Two plated-through vias are introduced to connect the resistor and stripline within the Wilkinson divider. A square groove with 3.4mm×2mm×0.286mm size is machined in the substrate together with the front metal layer printed on it, to facilitate the soldering of the coaxial connector and the middle feedline. The input port of the Wilkinson divider contains a transition from coaxial line to microstrip to stripline. A 200 Ω surface mount chip resistor is used to provide power-divider isolation.

The design guidelines for the proposed antenna structure are as follows.

### 2.1. Impedance-Matching Analysis

**Figure 3:**
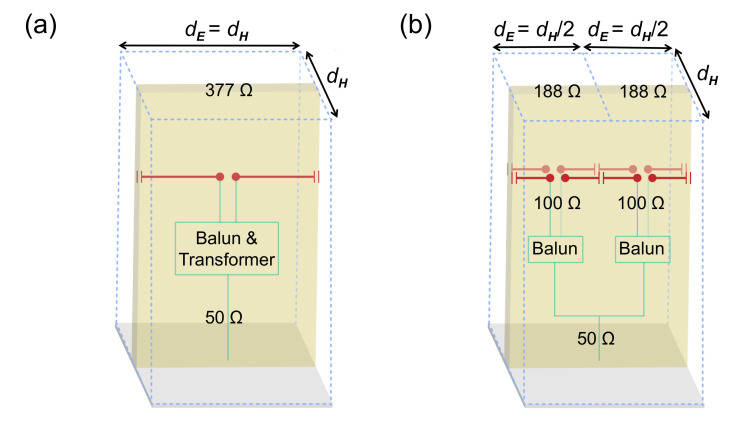
Presentation of the impedance-matching process. (**a**) Square unit cell with a single dipole; (**b**) Split unit cell with parallel dual dipoles.

The challenges of developing a practical tightly coupled array antenna include the balanced feeding and the impedance matching over a wideband and a large scan volume. For an infinite tightly coupled dipole array placed above a ground plane, the aperture impedance at broadside can be expressed as [29]

(1) $$R_{A}=\eta_{0}\frac{d_{E}}{d_{H}},$$

where $\eta_{0}$ is the free-space wave impedance, and $d_{E}$ and $d_{H}$ are the E- and H-plane element spacings, respectively. Thus, for most situations, the aperture impedance is about 377 Ω for an array with a square lattice ($d_{E}=d_{H}$). As shown in Figure 3a, when matching this high impedance to the standard 50 Ω interface, a wideband balun and a multi-section transformer are usually required.

In our design, two strategies are used to lower the aperture impedance. First, like the TCDA with integrated balun presented in [24], a dense sampling of the array in the E-plane ($d_{E}=\frac{1}{2}d_{H}$) is implemented, and thus the dipole impedance is correspondingly reduced by a factor of two, about 188 Ω. Second, using two sets of parallel-dipole structures again reduces the input impedance of each split unit cell by another factor of two, now about 100 Ω. Finally, the power combiner is used to transform two 100 Ω impedance to a 50 Ω coaxial connector. It can be seen that the antenna in this paper is designed without stepped feedlines, thus reducing the length of the feeding network and the loss. More importantly, this feeding approach is easily scaled to other frequency bands without much tuning. Figure 3b depicts a schematic diagram for this process.

**Figure 4:**
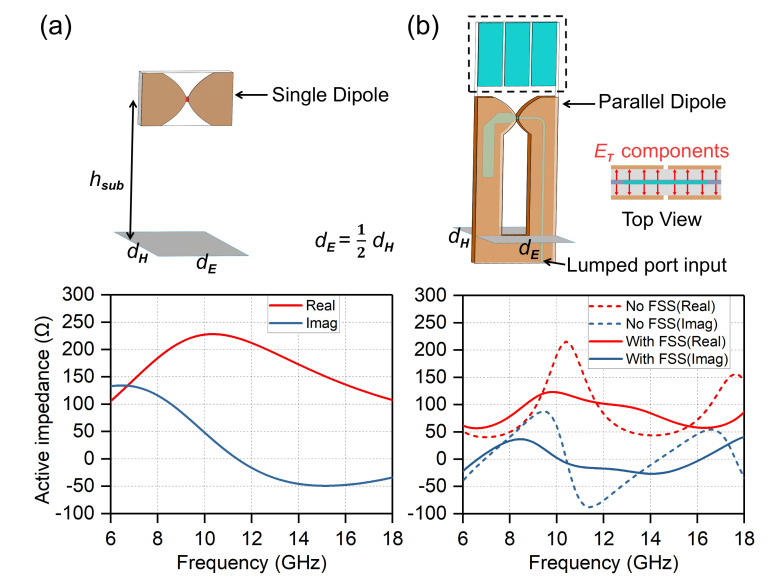
Active impedance analysis of the unit cell with periodic boundary condition using full-wave simulation software. (**a**) Single-dipole element; (**b**) Parallel-dipole element with a Marchand balun.

Figure 4 presents the preliminary design of the array element. A unit cell of the infinite single-dipole array fed by a lumped gap source is shown in Figure 4a. The gray rectangle represents the metal ground. The distance from the dipole to the ground is $h_{sub}=$ 5.5 mm, the dipole width is 2 mm, and $d_{E}=\frac{1}{2}d_{H}=$ 3.5 mm. The unit cell was simulated under periodic boundary conditions, which can indicate the infinite array performance. The simulated results indicate that the real active input impedance at the resonance point reaches up to 200 Ω, which is about half of the wave impedance of the free space. And the impedance curves change a lot over the observation frequency band, which are very detrimental to realize wideband impedance matching. Then one more dipole was added to the opposite side of the dielectric substrate, constituting a parallel-dipole form, as shown in Figure 4b. The E-plane element spacing is still halved, and the detailed dimensions keep the same with the single-dipole counterpart. A stripline-based Marchand balun is employed to feed this dipole pair. By comparing the simulated results, we can find that the average active input resistance drops to 100 Ω, though still with large fluctuations. The capacitive coupling introduced by the parallel combination of the dipole pair, cancels out the ground plane inductance at low frequencies. An FSS structure was further placed over the dipole element to improve matching. After that, the real part of the impedance flattens out near 100 Ω, and the imaginary part is stable around 0 Ω over the operating band.

**Figure 5:**
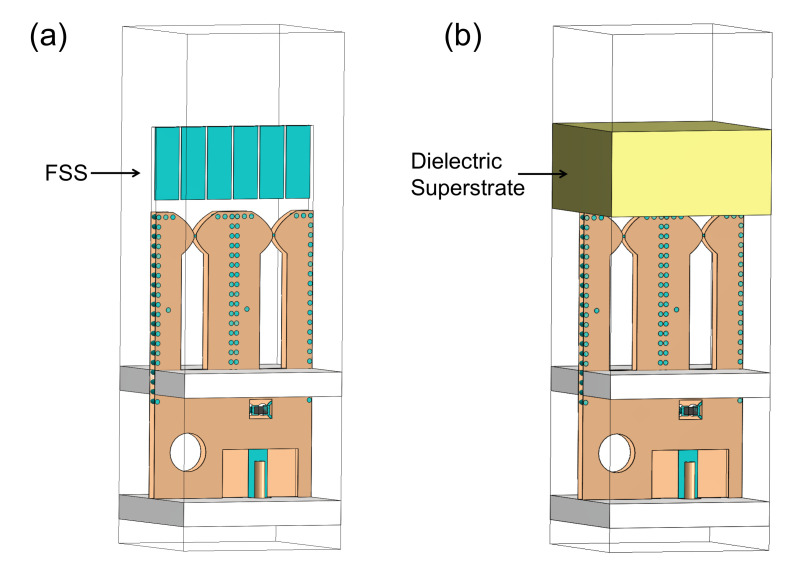
Unit cell of the proposed TCDA with (**a**) FSS and (**b**) equivalent dielectric superstrate.

**Figure 6:**
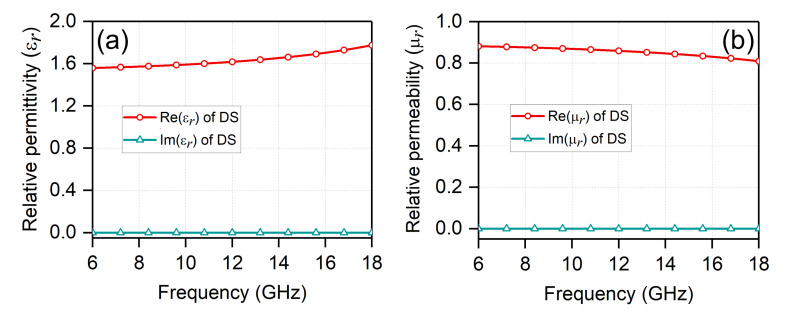
(**a**) Relative permittivity of the equivalent DS; (**b**) Relative permeability of the equivalent DS.

To reveal the nature of electromagnetic response of the FSS superstrate components, an equivalent dielectric analysis method is used [37]. It is well known that dielectric layers can compensate for the impedance variations during scanning, therefore improving the total scan volume of a phased array [21,38,39]. While the use of the bulky dielectric superstrates would increase the weight and cost of the array. The printed FSS superstrates, which are much lighter, can be equivalent to dielectric blocks to achieve the same functionality [32,33,34,35]. Figure 5b plots the unit cell of the proposed TCDA with equivalent dielectric superstrate (DS). The relative permittivity and relative permeability of the equivalent DS are shown in Figure 6. The real parts of relative permittivity and relative permeability are around 1.6 and 0.8, respectively. The imaginary parts of relative permittivity and relative permeability are about zero.

**Figure 7:**
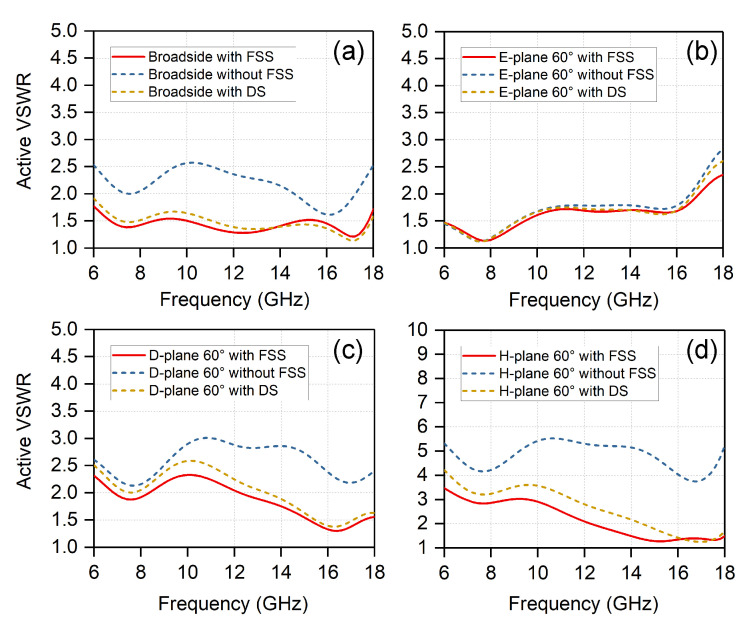
Comparisons of active VSWR when scanning to (**a**) broadside; (**b**) 60${}^{\circ }$ in the E-plane; (**c**) 60${}^{\circ }$ in the D-plane; and (**d**) 60${}^{\circ }$ in the H-plane.

The electromagnetic simulations of the unit cell with DS were carried out. Figure 7 compares the active VSWR of the infinite arrays without superstrates, with the FSS superstrate, and with equivalent DS at different scanning angles. Without FSS or DS, the active VSWR deteriorates when scanning to the broadside and 60${}^{\circ }$ in the H-/D-planes. The solid red curves and dashed yellow curves are very close, which indicates that the FSS superstrate and equivalent DS have similar capabilities at different scanning angles. In addition, the FSS superstrate is even more effective than the equivalent DS. The FSS superstrate is especially helpful for the H-plane scan, since the active VSWR is much higher when scanning to H-plane 60${}^{\circ }$ for the case of without FSS

**Figure 8:**
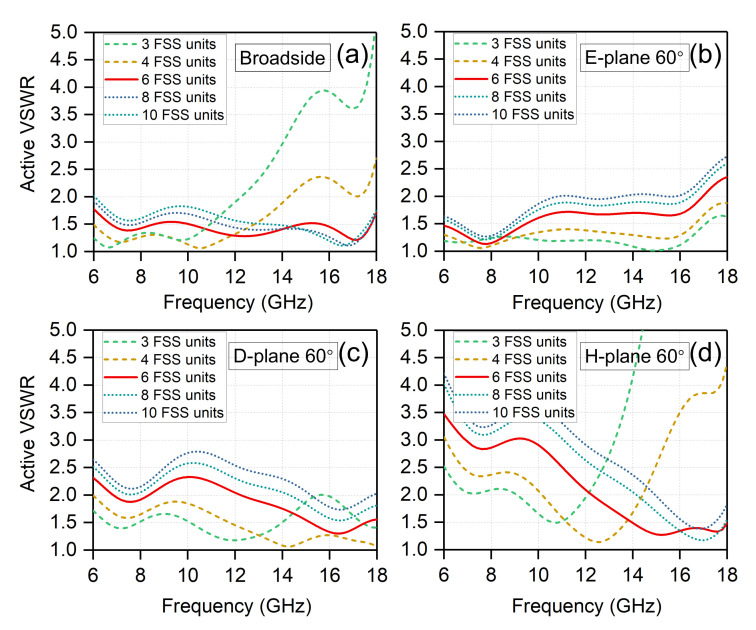
The impacts of the number of FSS units within an antenna unit cell on the active VSWR when scanning to (**a**) broadside; (**b**) 60${}^{\circ }$ in the E-plane; (**c**) 60${}^{\circ }$ in the D-plane; and (**d**) 60${}^{\circ }$ in the H-plane.

An FSS is a periodic structure, which is basically an assembly of identical elements arranged in a one or two-dimensional array. In our case, the total number of the FSS units in a 8 × 8 antenna array is 48 × 48, which are as a superstrate shared by all the antenna elements, as shown in Figure 1a. There are six FSS units in one antenna unit cell. The width of the FSS unit is much less than $\lambda_{h}$, about $\lambda_{h}/12$, thus the FSS superstrate always operates below its resonance frequency in the whole band. The antenna unit cell with different numbers of FSS units were simulated. The gaps between the FSS units remained unchanged. Figure 8 shows the impacts of the number of FSS units on the active VSWR when scanning in different planes. As shown in Figure 8, when the number of the FSS units decreases from 6 to 3 or 4, the active VSWR gets better when scanning to 60${}^{\circ }$ in the E-/D-planes, but the active VSWR deteriorates seriously when scanning to broadside and 60${}^{\circ }$ in the H-plane at high frequencies. The width of the FSS unit will increase as the number decreases, approaching $\lambda_{h}/2$, thus this will affect the high-frequency performance first. Therefore, the number of FSS units should not be too small. When the number of the FSS units increases from 6 to 8 or 10, the active VSWR gets worse slightly. It should be noted that increasing the number may achieve the same good performance when some optimizations are carried out. However, this will result in many small metallic sheets, which is undesired during machining process.

### 2.2. Undesired Resonances Mitigation

The mitigation of undesired resonances that may exist in the array is also a practical problem. For the element spacings in the E-/H-planes are both less than a half-wavelength, the common-mode resonances resulted from the unbalanced feeding at these two principal planes will not appear. While as observed in [29], a strong vertical field distribution along the diagonal planes is possible to occur. This common-mode resonance comes out when the diagonal feeding path reaches a half-wavelength. In our design, the vertical feed lines are well shielded for the use of stripline-based feeding network. Hence, the potential common-mode resonances in these directions can be avoided. Moreover, compared with microstrip lines, striplines can better prevent electromagnetic leakage and interference, thus supporting denser component layout, especially for higher frequencies.

**Figure 9:**
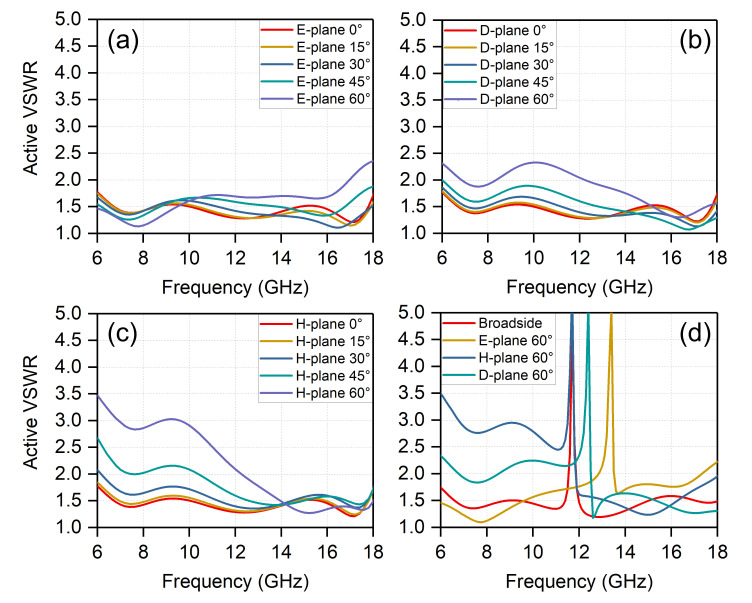
(**a**–**c**) Simulated active VSWR of proposed array in the E-/H-/D-planes at five different scanning angles; and (**d**) its contrast without plated-through vias.

**Figure 10:**
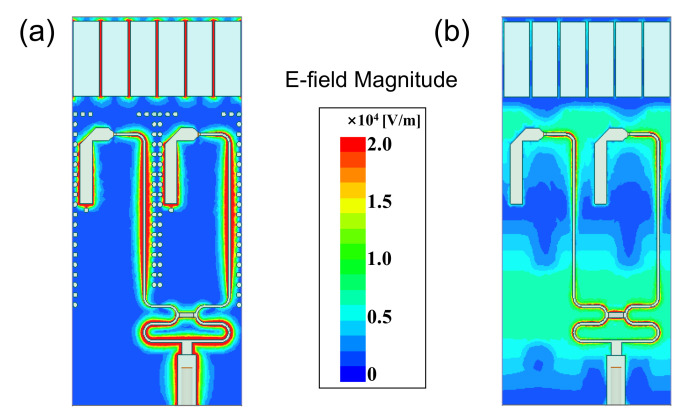
Simulated E-Field magnitude distribution of the proposed array at 11.7 GHz when scanning to broadside. (**a**) With plated-through vias (non-resonance); (**b**) Without plated-through vias (resonance).

Other possible types of resonances include the direct parasitic coupling between the adjacent baluns in scanning, and the parallel plate modes between the front and back PCB layers. Plated-through vias are placed at the feedline edges to circumvent these issues, depicted in Figure 1. Figure 9 presents the simulated active VSWR of the final configuration shown in Figure 1. Without vias, several reflection anomalies come out at different scanning angles; see Figure 9d. We examined the fields within the unit cell for 11.7 GHz at broadside, which is corresponding to a reflection anomaly. As seen in Figure 10b, the electric fields occupy the entire dielectric substrate. For comparison, Figure 10a shows the electric fields for the unit cell with vias at the same frequency, which are well confined inside the feedline. This indicates the effectiveness of the plated-through vias.

As shown in Figure 9a–c, the active VSWR of the proposed infinite array maintains <2.5 when scanning up to 60${}^{\circ }$ in the E-/D-planes, and maintains <3.5 when scanning up to 60${}^{\circ }$ in the H-plane across the entire 3:1 frequency band. The impedance matching for beam scanning in the E-plane is better than that in the H-plane, and the D-plane results follow an approximate average of the E- and H-planes.

### 2.3. Unit Cell Gain

**Figure 11:**
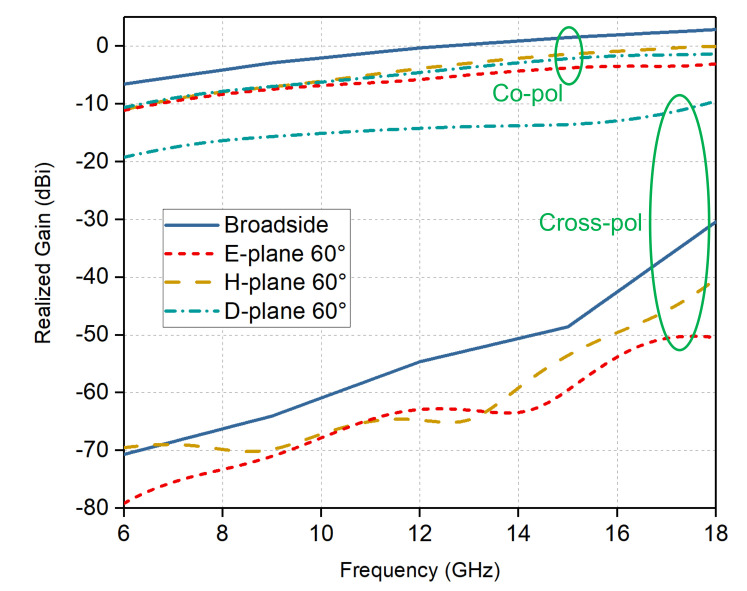
Simulated realized gains (co-polarization and cross-polarization) per unit cell of the proposed array for broadside and wide-angle scanning cases in all planes.

Figure 11 plots the simulated co-polarization and cross-polarization level per unit cell of the infinite array for scanning to broadside and 60${}^{\circ }$ in all planes. The co-polarized gain gradually increases with frequency and decreases with scanning angle. For 60${}^{\circ }$ scanning in the H-/D-planes, the co-polarized gain is about 3.5dB lower than that of broadside across the entire frequency band. It can be seen that the scan loss at high-frequency band in the E-plane is a little higher but within acceptable limits. The cross-polarization performance of the design is superior, which is one more benefit of the parallel dipoles. The cross-polarization is defined following Ludwig’s third definition [40]. The simulated cross-polarization is at least {−33 dB, −47 dB, −40 dB} lower than the co-polarization at {broadside, E-plane 60${}^{\circ }$, H-plane 60${}^{\circ }$}, respectively. Compared with the typical single-dipole TCDA, which has −20 dB cross-polarization level at broadside and when scanning in principal planes, the parallel-dipole design has a significant improvement. Here the proposed array employs the similar cross-polarization suppression mechanism with the balanced antipodal Vivaldi antenna [41,42], but with better cross-polarization performance due to more symmetrical structure. The strong transverse E-field components ($E_{T}$) perpendicular to the dipole arms, which is usually occurred in a conventional single-layered dipole array, can be counteracted due to the double-sided radiating structures, thus leading to better cross-polarization characteristics, as shown in the top view in Figure 4b. It is worth noting that the cross-polarization suppression mechanism is more effective in the principal planes. While the D-plane cross-polarization levels keep well, remaining below −8.5 dB at 60${}^{\circ }$ over the whole frequency band. This generally agrees with the ideal linearly polarized dipole apertures where the cross-polarization level is −9.5 dB at 60${}^{\circ }$ [18].

## 3. Fabrication and Measurement

**Figure 12:**
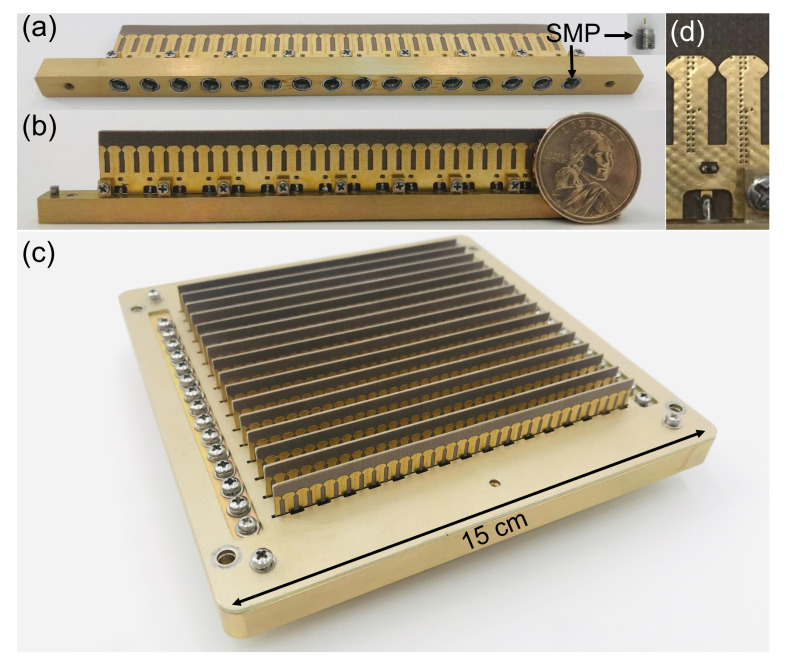
Fabricated 16 × 16 prototype and detailed illustrations for antenna elements. (**a**) Bottom view of a subarray; (**b**) Front view of a subarray; (**c**) 16 × 16 prototype; (**d**) Enlarged view of the prototype.

After indication of good performance in an infinite array environment, a 16 × 16 prototype array was fabricated and measured. As depicted in Figure 12, its overall dimensions are 15cm×15cm×2cm. The module design is adopted to make maintenance easy. The array includes 16 vertically placed PCBs, and each is screwed to a strip metal base. The upper ground plane contains a series of slots, and thus each board can be inserted vertically from the back of the metal framework. Two locating pins on the edges of the strip metal base are used to ensure the proper spacing between the boards. The antenna elements are fed by the SMP connectors embedded in the strip metal base. It should be noted that some of the plate-through vias are removed for the convenience of machining, as shown in Figure 12d, which does not affect the desired performance of the array.

### 3.1. Array Impedance Results

**Figure 13:**
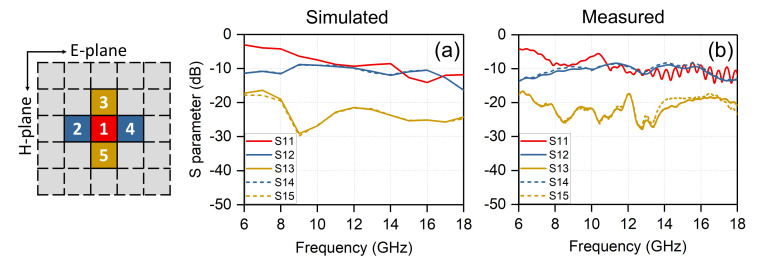
Simulated and measured S-parameter for the selected ports of the prototype array.

To evaluate the impedance-matching results of the array, the active VSWR measurements of the central element (8, 8) were conducted using an Agilent vector network analyzer. The reflection and transmission coefficients of the central element with all other surrounding elements were collected, while all other ports are matched to 50 Ω. The active VSWR of an element (m,n) within an array can be expressed as,

(2) $$\Gamma_{mn}\left(\varphi_{0},\theta_{0}\right)=\sum_{p=1}^{M}\sum_{q=1}^{N}S_{mn,pq}e^{-jk_{0}\left[\left(m-p\right)d_{x}sin\theta_{0}cos\varphi_{0}+\left(n-q\right)d_{y}sin\theta_{0}sin\varphi_{0}\right]},$$

where ($\varphi_{0},\theta_{0}$) is the array scan direction, $S_{mn,pq}$ is the s-parameter between the element mn and pq, $k_{0}$ is free-space wavenumber, M,N are the number of elements, $d_{x},d_{y}$ are the lattice spacings in the E- and H-planes, respectively. To gain insight to the coupling results between the unit cells of this kind of tightly coupled array, Figure 13 plots the simulated and measured reflection and transmission coefficients of the central port with four adjacent ports. The curves of S13 and S15 coincide perfectly, and the same is true for S12 and S14 curves, which are due to the symmetry of the array. The trends of simulated and measured results show a good consistency, excepted for some valleys in the measured curves. Machining and measurement errors may lead to this phenomenon. The red curves represent the reflection coefficients S11. We can find that the amplitudes of S11 are higher than −10 dB in the low band, while the active VSWR is good at broadside (see Figure 9). This just indicates the operating principle of tightly coupled arrays, which is working by using the strong mutual coupling between units, especially for the low frequencies. The yellow curves represent the transmission coefficients S13 and S15, which indicate the inter-element coupling in the H-plane. The blue curves indicate the inter-element coupling in the E-plane. It can be seen that the E-plane coupling is stronger than that of H-plane. As it is well known that the coupling strengths decrease as the distance increases, the coupling results with other ports are not shown.

**Figure 14:**
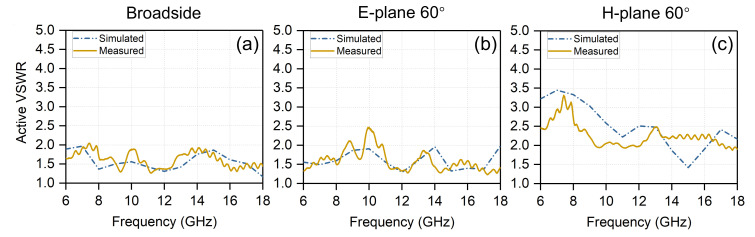
Simulated and measured active VSWR versus frequency and scan angle for the embedded element within the proposed array. (**a**) Broadside; (**b**) E-plane 60${}^{\circ }$; (**c**) H-plane 60${}^{\circ }$.

The simulated and measured active VSWR of the central element (8, 8) is shown in Figure 14. The active VSWR of the proposed array maintains <2.5 when scanning up to broadside and 60${}^{\circ }$ in the E-plane, and maintains <3.5 when scanning up to 60${}^{\circ }$ in the H-plane. The measured and simulated results of the proposed finite array largely follow the same trends. The rapid ripples may be introduced by the testing cables.

### 3.2. Far-Field Radiation Characteristics

**Figure 15:**
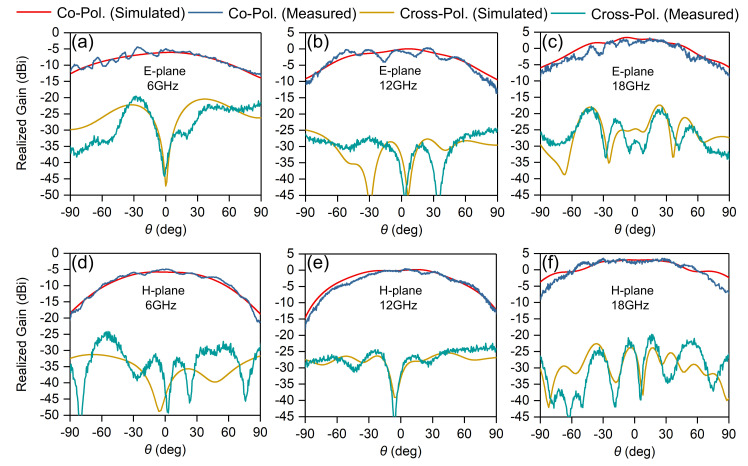
Comparisons of measured and simulated embedded element patterns for the central element (8, 8) of the proposed array. (**a**) E-plane @ 6 GHz; (**b**) E-plane @ 12 GHz; (**c**) E-plane @ 18 GHz; (**d**) H-plane @ 6 GHz; (**e**) H-plane @ 12 GHz; (**f**) H-plane @ 18 GHz.

The embedded element patterns of the central element (8, 8) within the array were measured in an anechoic chamber and the results compared with simulations, thus we can gain insight into behaviors of the element in the presence of other radiators. Figure 15 shows the measured and simulated embedded element patterns of selected frequencies in principal planes, including 6 GHz, 12 GHz, 18 GHz. Measured and simulated co- and cross-polarized patterns are in good agreement. Small ripples varied within 2dB can be observed in the measured co-polarized patterns, which are mainly due to the small, finite size of the array and will decrease as the array size increases.

**Figure 16:**
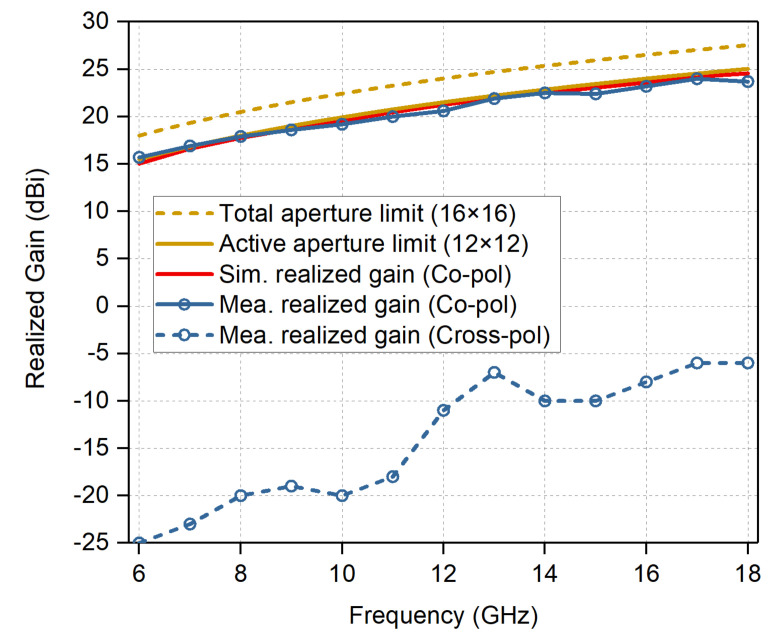
Measured co-polarized and cross-polarized realized gains at broadside of the prototype array, along with the simulated gains and the theoretical aperture limits.

Of particular interest is the fully excited array scanning patterns. The measurement was conducted following the unit excitation active element pattern method [43]. The active part of the aperture was chosen to be 12 × 12, with edge elements terminated with matched loads to mitigate edge reflection effect. The measured and simulated broadside gains versus frequency are plotted in Figure 16, along with the theoretical gain limits for the active aperture and the total aperture. The ideal aperture limit is calculated using 4π*A*/$\lambda_{0}^{2}$, where *A* is the aperture area, and $\lambda_{0}$ is the wavelength in free space. The dashed yellow curve is the total aperture limit of the 16 × 16 array. The solid yellow curve is the active aperture limit of the active 12 × 12 array within the 16 × 16 array. The red curve is the simulated broadside gain of the active 12 × 12 aperture. As can be seen, the simulated and measured data agree well for the whole frequency band. The measured co-polarized gains are very close to the active aperture limits, which indicates a high aperture efficiency. The measured broadside cross-polarized gains of the array remain at least 29 dB lower than the co-polarized gains within the operation band. The measured results are a little higher than the simulated cross-polarization level, probably resulting from the measurement precision limit of the anechoic chamber and the misalignments of the array and the feeding antenna.

**Figure 17:**
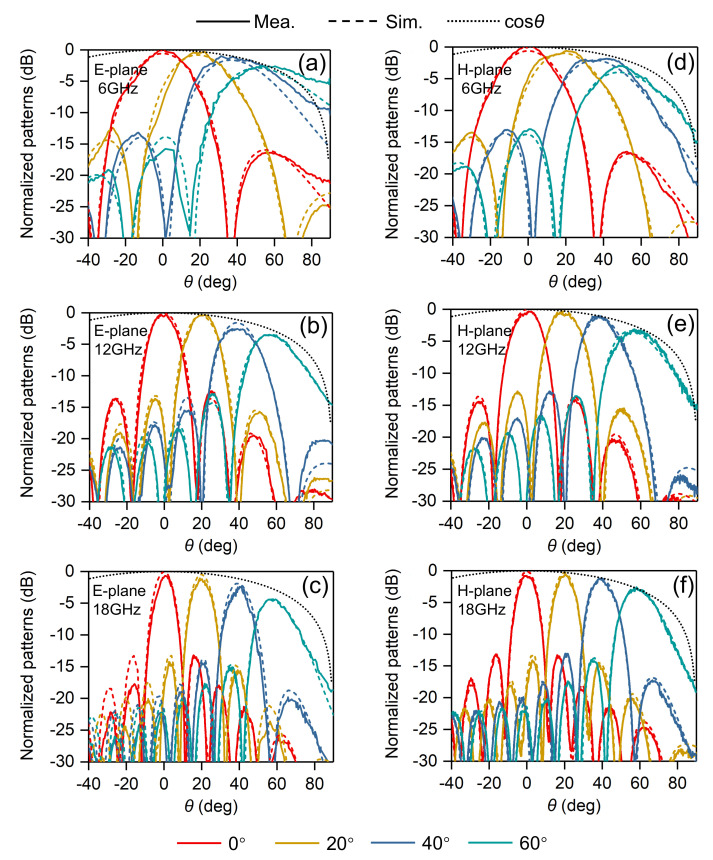
Measured and simulated normalized radiation patterns for 0${}^{\circ }$, 20${}^{\circ }$, 40${}^{\circ }$, and 60${}^{\circ }$ scans in the principal planes. (**a**) E-plane @ 6 GHz; (**b**) E-plane @ 12 GHz; (**c**) E-plane @ 18 GHz; (**d**) H-plane @ 6 GHz; (**e**) H-plane @ 12 GHz; (**f**) H-plane @ 18 GHz.

The scanning ability of the array across the two principal planes at 6 GHz, 12 GHz and 18 GHz are displayed in Figure 17. The patterns are normalized to the peak broadside gains. The measured patterns are in good agreement with the simulated ones, except for the tiny ripples within the measured patterns. The black cosθ curve indicates the optimal scan loss for a large planar array.

The normalized gains at different scanning angles are roughly equal to the value of broadside gain multiplying cosθ for most of the cases. As shown in Figure 17d, compared with the ideal cosθ curve, a larger gain drop is observed for H-plane 60${}^{\circ }$ scan at 6 GHz. This is mainly due to the impedance mismatch of the antenna. As we can see from Figure 9c, the H-plane active VSWR levels for 60${}^{\circ }$ scan in the low band are relatively high. Similar phenomenon is observed for E-plane 60${}^{\circ }$ scan at 18 GHz. Compared with the ideal cosθ curve, a relatively large gain drop is shown; see Figure 17c. The reason for this phenomenon is different from that of H-plane, since the E-plane scanning VSWR is good. This is mainly due to the use of the Wilkinson divider. The two units are combined along E-plane with a Wilkinson power divider. For large scan angles in the E-plane, the phase difference between the two combined unit cells will be increasing with scan angle and frequency. Therefore, there will be a certain amount of power coupling/dissipating in the isolation resistor of the Wilkinson. The beam pointing deviations at low frequencies for large scan angles will be alleviated when the array size is large enough. In all scan cases, the sidelobes are well below −12 dB.

The performance of the proposed array are compared with previous works on TCDA antennas, as shown in Table 1. In addition to the simple and effective impedance-matching process, the results show the merit of our array in large scan sector and excellent polarization purity. It should be noted that further improvements can be made on the proposed array. Wider working bandwidth can be realized by enhancing the inter-element capacitive coupling. Furthermore, the proposed TCDA topology can be extended to a dual-polarized configuration, as dual-linear or dual-circular polarizations are of particular interest in many UWB array applications. An “egg-crate” construction [24] can be employed to construct the dual-polarized one, in which a second orthogonal set of elements should be added to the single-polarized array. Clearly, the vertical printed boards cannot cross at the feed centers, otherwise the Wilkinson dividers will be split. Thus, the boards can only cross at the junctions between the unit cells, yielding elements with offset phase centers. Several modifications have to be made on the single-polarized elements, including: (1) Removing the plate-through vias located at the junctions; (2) Cutting partial slots on the boards to avoid the overlaps of the orthogonal elements; (3) Adjusting the widths of the FSS units to avoid overlapping with the orthogonal elements.

## 4. Conclusions

This paper presents an ultrawideband, wide scanning TCDA with low cross-polarization level, light weight and compact size. The tightly coupled dipoles employ parallel symmetrical structures, which can reduce the high aperture impedance by a factor of almost four and cancel out the strong transverse E-field components perpendicular to the dipole arms. The proposed antenna employs a simple feeding structure, eliminating the need for the bulky dielectric superstrate or the multi-section stepped transformer. And it is able to operate over a 3:1 bandwidth (6−18 GHz) while scanning to 60${}^{\circ }$ in the E-/D-planes for active VSWR < 2.5 and 60${}^{\circ }$ in the H-plane for active VSWR < 3.5. A 16 × 16 array prototype is fabricated and the central 12 × 12 elements are measured to validate the design. The realized gains and radiation patterns show good agreement with simulations.

## Figures and Tables

**Table 1:** Comparison of array performance with the reported references.

Ref.	Feeding Type (Antenna Form)	Bandwidth	Cross-Pol. @Broadside	Scanning Range	VSWR	Polarization
[3]	TCDA-IB	2.7:1	−15 dB	E, ±45${}^{\circ }$	<2.2	Single-polarized
	(Single dipole)	(2.2–6 GHz)				
[13]	Microstrip	2.2:1	-	E, ±50${}^{\circ }$	<2.0	Single-polarized
	(Connected slots)	(6.5–14.5 GHz)		H/D, ±50${}^{\circ }$	<2.0	
[18]	Coaxial cable	1.6:1	−18 dB	E, ±70${}^{\circ }$	<2.0	Single-polarized
	(Planar dipole)	(8–12.5 GHz)		H/D, ±60${}^{\circ }$	<2.0	
[23]	Coaxial cable	15:1	−25 dB	E, ±60${}^{\circ }$	-	Dual-polarized
	(Planar dipole)	(0.13–2 GHz)		H/D, ±60${}^{\circ }$	-
[24]	TCDA-IB	7.35:1	−15 dB	E, ±45${}^{\circ }$	<2.7	Single-polarized
	(Single dipole)	(0.68–5 GHz)		H/D, ±45${}^{\circ }$	<2.7	
[29]	PUMA	3:1	−15 dB	E, ±45${}^{\circ }$	<2.5	Dual-polarized
		(7–21 GHz)		H/D, ±45${}^{\circ }$	<3.0	
[30]	PUMA	6:1	−20 dB	E, ±60${}^{\circ }$	<2.0	Dual-polarized
		(3.53-21.2 GHz)		H/D, ±60${}^{\circ }$	<3.8	
[33]	TCDA-IB	5:1	−15 dB	E, ±60${}^{\circ }$	<3.0	Dual-polarized
	(Planar dipole)	(0.4–2 GHz)			
[36]	TCDA-IB	5.5:1	−21 dB	E, ±70${}^{\circ }$	<3.2	Single-polarized
	(Single dipole)	(0.8–4.38 GHz)		H, ±55${}^{\circ }$	<3.2	
This	TCDA-IB	3:1	−29 dB	E/D, ±60${}^{\circ }$	<2.5	Single-polarized
Work	(Parallel dipole)	(6-18 GHz)		H, ±60${}^{\circ }$	<3.5	
